# Covid-19 vaccination priorities defined on machine learning

**DOI:** 10.11606/s1518-8787.2022056004045

**Published:** 2022-03-11

**Authors:** Renato Camargos Couto, Tania Moreira Grillo Pedrosa, Luciana Moreira Seara, Carolina Seara Couto, Vitor Seara Couto, Karla Giacomin, Ana Claudia Couto de Abreu

**Affiliations:** I Fundação Lucas Machado Faculdade de Ciências Médicas de Minas Gerais Belo Horizonte MG Brasil Fundação Lucas Machado. Faculdade de Ciências Médicas de Minas Gerais. Belo Horizonte, MG, Brasil; II Instituto de Acreditação e Gestão em Saúde Departamento de Tecnologia da Informação Belo Horizonte MG Brasil Instituto de Acreditação e Gestão em Saúde. Departamento de Tecnologia da Informação. Belo Horizonte, MG, Brasil; III Instituto de Assistência Médica ao Servidor Público Estadual de São Paulo. Hospital do Servidor Público Estadual Programa de Residência Médica São Paulo SP Brasil Instituto de Assistência Médica ao Servidor Público Estadual de São Paulo. Hospital do Servidor Público Estadual. Programa de Residência Médica. São Paulo, SP, Brasil; V Centro Internacional de Longevidade Belo Horizonte MG Brasil Centro Internacional de Longevidade. Belo Horizonte, MG, Brasil

**Keywords:** COVID-19 vaccines, supply & distribution, Immunization Programs, Health Priorities, Machine Learning

## Abstract

**OBJECTIVE:**

Defining priority vaccination groups is a critical factor to reduce mortality rates.

**METHODS:**

We sought to identify priority population groups for covid-19 vaccination, based on in-hospital risk of death, by using Extreme Gradient Boosting Machine Learning (ML) algorithm. We performed a retrospective cohort study comprising 49,197 patients (18 years or older), with RT-PCR-confirmed for covid-19, who were hospitalized in any of the 336 Brazilian hospitals considered in this study, from March 19th, 2020, to March 22nd, 2021. Independent variables encompassed age, sex, and chronic health conditions grouped into 179 large categories. Primary outcome was hospital discharge or in-hospital death. Priority population groups for vaccination were formed based on the different levels of in-hospital risk of death due to covid-19, from the ML model developed by taking into consideration the independent variables. All analysis were carried out in Python programming language (version 3.7) and R programming language (version 4.05).

**RESULTS:**

Patients’ mean age was of 60.5 ± 16.8 years (mean ± SD), mean in-hospital mortality rate was 17.9%, and the mean number of comorbidities per patient was 1.97 ± 1.85 (mean ± SD). The predictive model of in-hospital death presented area under the Receiver Operating Characteristic Curve (AUC - ROC) equal to 0.80. The investigated population was grouped into eleven (11) different risk categories, based on the variables chosen by the ML model developed in this study.

**CONCLUSIONS:**

The use of ML for defining population priorities groups for vaccination, based on risk of in-hospital death, can be easily applied by health system managers

## INTRODUCTION

Brazil is an upper middle-income country with 213 million inhabitants and a large territorial area, an aggravating factor to the unprecedented pressure placed by the covid-19 pandemic on healthcare systems countrywide^1^. Among the many issues faced by the country is the increased hospitalization rates, as well as increased demand for intensive care unit (ICU) beds, advanced respiratory support, and trained health professionals^2^.

The first confirmed case of covid-19 in Brazil was reported on February 26^th^, 2020. A year and a half later, the country accounts for 21,810,855 cases and 607,824 deaths^2^.

A global equitable access to the covid-19 vaccine, mainly to protect health professionals and individuals at high risk, is the only way to mitigate the pandemic’s impact on the economy and public health^3^.

There is a severe shortage of vaccines and hospital resources worldwide, as well as huge imbalance in vaccine distribution between rich and poor countries. High-income countries currently have a total of 17.8 billion vaccine doses, 6.8 billion of which are reserved; whereas low-income countries have only 394.5 million doses^4,5^. This difference makes the determination of priorities even more urgent in countries with low resource availability.

Vaccinating the population, especially those at risk of death, is necessary to help minimizing the consequences of such an unequal distribution of vaccines and resources.

This study sought to define covid-19 vaccination priorities based on risk of in-hospital death by using the developed ML model, which was based on variables such as age, sex, and chronic health conditions.

## METHODS

**Figure 1:**
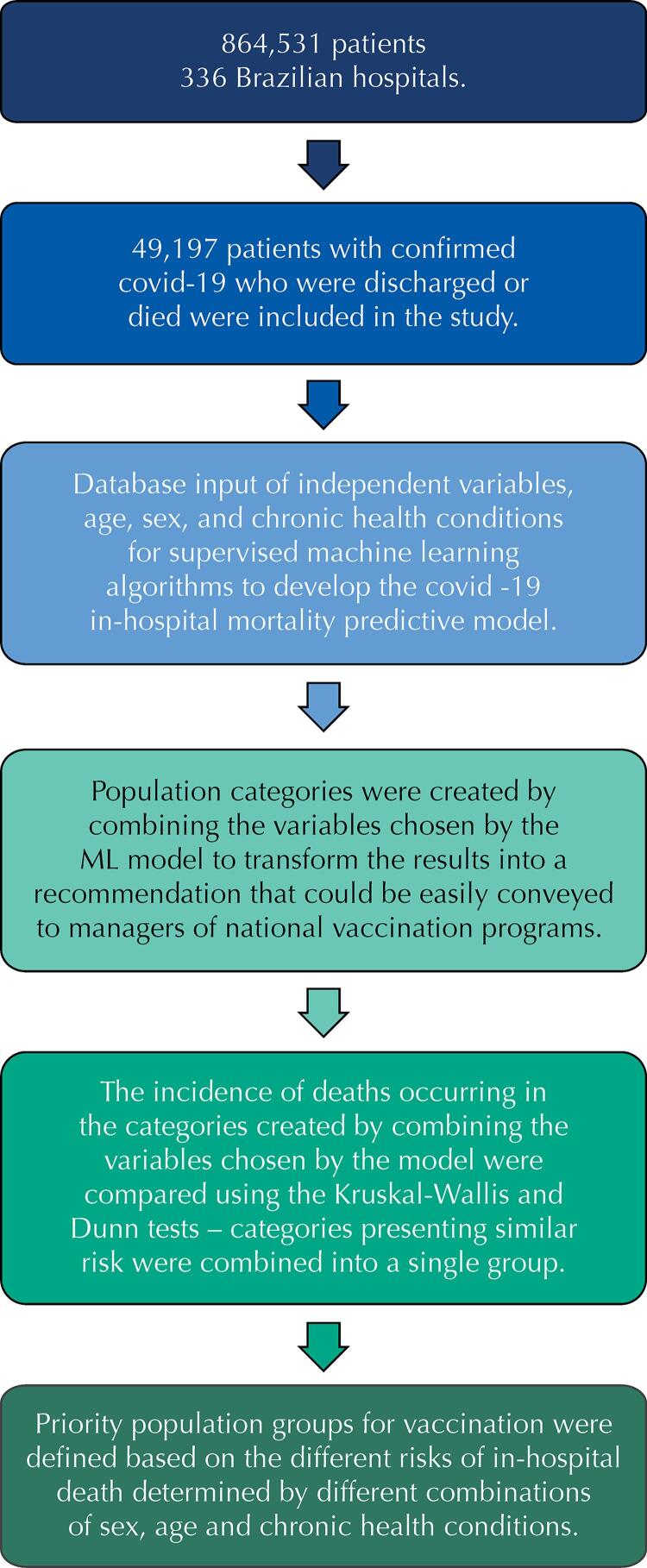
Flowchart describing the development of the predictive model applied to in-hospital death due to covid-19, and the definition of priority groups for vaccination based on age, sex and chronic health conditions.

In summary, the steps shown in Figure 1 were followed for the development of the predictive model through machine learning, applied to in-hospital death by covid-19, and the definition of priority groups for vaccination based on age, sex, and chronic health conditions:

- Among the 864,531 patients admitted to 336 public and private Brazilian hospitals, during the study period, only the 49,197 patients with RT-PCR-confirmed for covid-19, who were discharged or died, were included in the study.
- An anonymized database was created with the patients included in this study, where the independent variables were age, sex, and 179 chronic health conditions, and the outcome variable was the occurrence or not of death. For the purpose of our study, chronic health conditions comprise comorbidities that are defined as the coexistence of another medical condition alongside covid-19 infection at the time of the patients’ hospitalization or the use of external devices and interventions to keep the patient alive, such as tracheostomy, ventilatory support, and dialysis. This database was the input for the development of the covid-19 in-hospital mortality predictive model, with the use of the supervised machine learning algorithms.
- The ML model chose the features to predict death, and population categories were created combining the main features chosen by the ML model.
- The incidence of death in the categories created was compared using the Kruskal-Wallis and Dunn statistical tests – categories with similar risk were gathered into a single group.
- Priority population groups for vaccination were created to turn the results into a recommendation that could be easily conveyed to national immunization program administrators. These groups were defined based on the different risks of in-hospital death determined by different combinations of sex, age, and chronic health conditions.

### Study Design and Participants

A retrospective cohort study was conducted with all patients, 18 years of age or older, who required hospitalization due to covid-19 infection – confirmed by positive result in polymerase chain reaction test applied to nasopharyngeal sample – and who died or were discharged, in any of the 336 hospitals in the Brazilian public and private healthcare system considered in this study, from March 19^th^, 2020, to March 22^nd^, 2021.

The study adopted anonymous convenience sample extracted from the DRG Brasil^®^ database, which is used by Brazilian public and private hospitals for managerial purposes. Data collection was carried out by nurses trained in medical coding, who were exclusively dedicated to this function and fully read the medical records of all patients after hospital discharge or death, inserting their data in DRG Brasil^®^ software. Diagnoses were classified based on ICD-10. The coding team was supervised by a support team, which, in turn, was supervised by authors 1 and 2 of this study, for data quality assurance purposes.

Acute complications due to covid-19 infection were excluded from the database, as well as chronic health conditions with less than 30 occurrences. This process resulted in dataset comprising of 181 independent variables such as age, sex, and 179 groups of chronic health conditions, whereas the outcome comprised hospital discharge or in-hospital death. Chronic health conditions were grouped into large categories comprising similar ICD-10 sets associated with the affected physiological system.

### Outcomes

The primary outcome was in-hospital death or discharge.

#### Machine Learning model development

The dataset was used as input for the supervised machine learning algorithms to develop the in-hospital death predictive model of covid-19 infected patients.

The predictive model development took into consideration 2 main goals^6^:

- Selecting the best model: estimating the performance of different models in order to select the best one.
- Model evaluation: estimating the prediction error (generalization error) of the selected model based on new data.

We used three ML algorithms to develop the predictive model – Random Forest, XGBoost, and Logistic Regression – and their performance was evaluated based on the Area Under the Receiver Operating Characteristic Curve (AUC ROC).

To select the best model we split the database into two parts, training data (70%) and testing data (30%). Training data were used for learning (the algorithm learns from the data, which contains the correct answer) and the test data was used for the second purpose mentioned above, which is to evaluate the model’s performance and generalization error on new data.

For the training step, we used the K-fold cross validation method^7^. This procedure has a single parameter k, which refers to the number of groups the training dataset should be divided into for training and validation purposes. One way to use this technique is to randomly divide the training set into k parts of equal size: k-1 parts are used to adjust the model, whereas the k^th^ part is used to estimate the model’s performance. The process continues until all parts have participated in both the training and validation processes – this procedure results in k performance estimates. The most common values used for k range from 5 to 10. We used k = 6 in all tested models, as higher k values did not result in better performances, but in longer processing time.

For the tree-based algorithms (Random Forest and XGBoosting), the algorithms’ hyperparameters were optimized during the cross-validation process and those that resulted in the best models were selected. The hyperparameters used for the XGBoost algorithm comprised learning rate – it determines the step size in each iteration as the model is optimized towards the goal (0.01, 0.05, 0.1, 0.2, and 0.25), max_depth – the maximum depth per tree (5, 10, 15, 20, 30), and n_estimators – The number of trees in final model (500, 1,000 and 2,000). The hyperparameters used for Random Forest were max_depth (15, 30, 50), n_estimators (100, 200, 500), and max_features – number of randomly selected predictors as candidates in each division of the decision trees (3, 6, 10)^7^.

Since logistic regression does not have hyperparameters, it was adjusted to training data once, based on the stepwise procedure.

AUC ROC were determined in each of the six cross-validation cycles and their respective confidence intervals (CI), calculated using the Delong method^8^ (95%CI). Subsequently, the selected model was applied to test data in order to assess its prediction error in future observations, also based on AUC ROC. The mean AUC ROC values recorded for all three algorithms were statistically compared to each other through Friedman’s Test, which was followed by Mann-Whitney Post-hoc test (at 5% significance level) – the one presenting the best performance was selected.

A calibration curve was also built to assess the predictive ability of the selected model. Calibration diagrams built based on the likelihood (generated by the predictive model) of a given event to take place enabled evaluating the model’s ability to make predictions. Calibration diagram is a linear graph representing the relative frequency of what was observed (axis y) versus the likely frequency predicted by the model (axis x), which enables comparing the curve generated by the model’s predictions to a standard curve; thus, it illustrates the model’s prediction performance. Predicted likelihoods are divided into a fixed number of intervals along axis x. Then, the number of events (class = 1) of each interval is counted (e.g., the observed relative frequency). Finally, counts are normalized, and results are plotted as line graph^9^.

The SHAP (SHapley Additive Explanations) technique was used to select the most predictive variables of the developed model. SHAP values are an extension of SHapley values in the game theory. They describe the effects of variables on a model’s output, besides being defined as the contribution of a specific variable to a given prediction. The advantage of using SHAP values lies on the fact that they add interpretability to complex models^10^.

All analysis and figure generation processes were carried out in Python programming language (version 3.7).

## Statistical Analysis

Population categories were created by combining variables chosen by the ML model to enable the transformation of results into recommendations that could easily be conveyed to national vaccination programs’ managers.

The incidence of death among categories created by the combination of variables chosen by the ML model was compared, through Kruskal Wallis statistical test, with the Dunn Post-hoc test – categories presenting similar risk were gathered in a single group.

We used medians and interquartile ranges (IQRs) or means and standard deviations (SDs) to summarize continuous variables, and calculate frequencies and proportions for categorical variables. Variables in the final model with a *p*-value of less than 0.05 were considered statistically significant.

All statistical analysis were performed in R programming language (version 4.05).

This study was approved by the Ethics and Research Committee of the Medical Sciences School of Minas Gerais (CAEE: 29000819.0.0000.5134). It was classified as low-risk study, since it used anonymous convenience sample extracted from the DRG Brasil^®^ database, which is used by Brazilian public and private hospitals for managerial purposes. The study did not require participants to sign the informed consent form.

## RESULTS

In total, 864,531 hospital discharges or deaths took place within the 336 investigated hospitals throughout this study. A total of 49,197 patients with RT-PCR-confirmed for covid-19 infection were hospitalized and 33.5% of the investigated hospitalizations took place in the Brazilian public health system (SUS).

Hospitalized patients’ mean age was 60.5 ± 16.9 years (18 to 108 years) and most of them were men (55.6%). In addition, 24,127 patients (49% of hospitalized patients) were 60 years old or younger, 10,335 patients (21.0%) were in the age group of 61–70 years, 8,187 patients (16.6%) were in the age group of 71–80 years, 5,208 patients (10.6%) were in the age group of 80–90 years and 1,340 patients (2.7%) were older than 90 years (Table 1).

**Table 1:** Patients in each of the 17 groups of features chosen by the ML model: number, outcome (death) and incidence of death.

Chronic health conditions	Patients, n/N (%)	Death, n_d_/N (%)	Incidence of death, n_d_/n (%)^a^
Age Group			
≥ 90 years	1,685/49,197 (3.4)	856/49,197 (1.7)	856/1,685 (50.8)
≥ 80 < 90 years	5,583/49,197 (11.3)	2,243/49,197 (4.6)	2,243/5,583 (40.2)
≥ 70 < 80 years	8,490/49,197 (17.3)	2,370/9,197 (4.8)	2,370/8,490 (27.9)
≥ 60 < 70 years	10,355/49,197 (21.1)	1,848/49,197 (3.8)	1,848/10,355 (17.8)
≥ 50 < 60 years	9,355/49,197 (19.0)	897/49,197 (1.8)	897/9,355 (9.6)
≥ 40 < 50 years	7,550/49,197 (15.4)	388/49,197 (0.8)	388/7,550 (5.1)
≥ 30 < 40 years	4,792/49,197 (9.7)	179/49,197 (0.4)	179/4,792 (3.7)
≥ 18 < 30 years	1,387/49,197 (2.8)	42/49,197 (0.1)	42/1,387 (3.0)
Sex			
Female	21,875/49,197 (55.5)	3,958/49,197 (8.0)	3,958/21,875 (18.1)
Male	27,322/49,197 (44.5)	4,865/49,197 (9.9)	4,865/27,322 (17.8)
Obesity	6,891/49,197 (14.0)	1,410/49,197 (2.9)	1,410/6,891 (20.5)
Chronic renal failure with dialysis	1,224/49,197 (2.5)	906/49,197 (1.8)	906/1,224 (74.0)
Chronic renal failure without dialysis	2,278/49,197 (4.6)	978/49,197 (2.0)	978/2,278 (42.9)
Myocardial and valvular heart diseases, and arrhythmias	3,319/49,197 (6.7)	1,319/49,197 (2.7)	1,319/3,319 (39.7)
Chronic arterial hypertension	23,881/49,197 (48.5)	5,592/49,197 (11.4)	5,592/23,881 (23.4)
Diabetes mellitus	12,549/49,197 (25.5)	3,106/49,197 (6.3)	3,106/12,549 (24.8)
Neoplasms	1,607/49,197 (3.3)	651/49,197 (1.3)	651/1,607 (40.5)
Chronic respiratory diseases	4,195/49,197 (8.5)	1,065/49,197 (2.2)	1,065/4,195 (25.4)
Cerebrovascular disease and its sequelae	848/49,197 (1.7)	363/49,197 (0.7)	363/848 (42.8)
Degenerative diseases of the central nervous system	980/49,197 (1.9)	475/49,197 (0.9)	475/980 (48.5)
Psychiatric disorders	1,161/49,197 (2.4)	189/49,197 (0.4)	189/1,161 (16.3)
Thyroid disease	3,736/49,197 (7.6)	853/49,197 (1.7)	853/3,736 (22.8)
Anemias	603/49,197 (1.2)	250/49,197 (0.5)	250/603 (41.5)
Transplant recipients and patients depending on respiratory support equipment	401/49,197 (0.8)	139/49,197 (0.3)	139/401 (34.7)
Hemorrhagic hematologic disease	387/49,197 (0.8)	166/49,197 (0.3)	166/387 (42.9)
Patients without any of the 15 chronic health conditions listed above	15,812/49,197 (31.2)	1,185/49,197 (2.4)	1,185/15,812 (7.5)

Total N = 49,197 patients; n = number of patients; n_d_ = number of dead.

^a^ Comparing the incidence of death between age groups (Kruskal-Wallis test and post-hoc Dunn test) showed that all age groups are statistically different from each other (p-value < 0.05).

In-hospital mortality of covid-19 patients was 18.7% (8,823 patients). Such a mortality rate increased as the age groups encompassed older patients: the incidence of death in the group younger than 30 years and in the one older than 90 years was 2.7% and 51.2%, respectively (Table 1). Moreover, 16,627 patients, hospitalized due to RT-PCR-confirmed covid-19 infection (33.8%), required intensive care, while 9,411 (19.1%) required invasive mechanical ventilation.

Mean number of chronic health conditions per patient was 1.97 ± 1.85 (mean ± SD); 11,695 (23.8%) patients included in this study did not have chronic health conditions, whereas 4,293 patients (8.7%) had more than 5 chronic health conditions. The most frequent chronic health conditions were: hypertensive diseases (23,881 patients; 48.5%), diabetes mellitus (12,549 patients; 25.5%;), obesity (6,891; 14.0%), chronic respiratory diseases (4,195; 8.5%), thyroid diseases (3,736; 7.6%), myocardial and valvular cardiac diseases and arrhythmias (3,319; 6.7%;), chronic renal failure (2,278; 4.6%), and neoplasms (1,607; 3.3%) (Table 1).

The model based on the Random Forest algorithm has shown the worst performance among the tested models. The other two models, which were based on the XGBoost and Logistic Regression algorithms, did not show significant differences from each other. Mean AUC ROC recorded for the model generated with the Random Forest algorithm was 0.762 (CI: 0.749, 0.775); it was significantly different (*p* < 0.05) from areas calculated based on the other two algorithms. Mean AUC ROC recorded for the model based on the XGBoost algorithm was 0.803 (CI: 0.788, 0.818), whereas the mean AUC ROC recorded for the Logistic Regression model was 0.801 (CI: 0.790, 0.812). Learning rate = 0.2, max_depth = 20, and n_estimators = 500 were the best hyperparameters for XGBoost. On the other hand, the best hyperparameters for Random Forest were n_estimators = 500 and max_features = 6.

**Figure 2:**
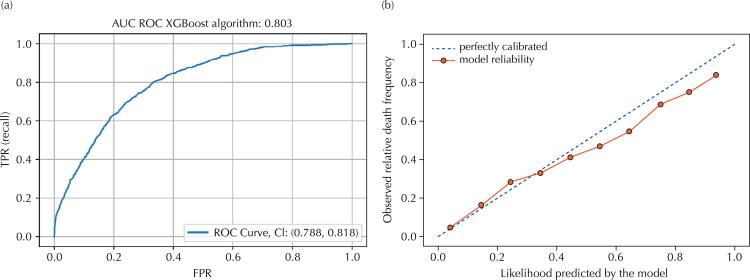
a) ROC curve for predictive model based on the XGBoost algorithm. b) Calibration curve for predictive model based on extreme gradient boosting (XGBoost) algorithm.

The XGBoost algorithm was selected due to its performance and greater robustness. Its calibration curve shows that the model performed reasonably well in predicting patients’ death. Predictions generated by the models and plotted on the graph were remarkably close to the reference curve. Figure 2 shows the ROC and the calibration curves for the model based on XGBoost algorithm. It is also possible to see in the figure the average area and their respective confidence intervals. The sensitivity and the specificity were 85% and 62.5%, respectively.

Legenda: AUC ROC: area under the receiver operating characteristic curve; XGBoost: extreme gradient boosting. TPR: true positive rate; FPR: false positive rate; CI: confidence interval

**Figure 3:**
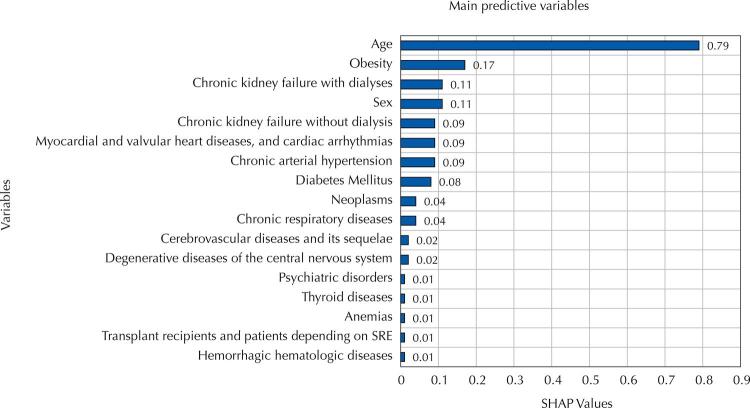
SHAP values recorded for the 17 main model features.

The SHAP method was applied to the model developed based on the XGBoost algorithm in order to define the most relevant features for incidence of death (Figure 3). The 17 most important independent variables for this model comprised age (SHAP +0.79), obesity (SHAP +0.17), chronic kidney failure with dialysis (SHAP +0.11), male sex (SHAP +0.11), chronic renal failure without dialysis (SHAP +0.09), myocardial and valvular diseases and cardiac arrhythmias (SHAP +0.09), chronic arterial hypertension (SHAP +0.09), diabetes mellitus (SHAP +0.08), neoplasms (SHAP +0.04), chronic respiratory diseases (SHAP +0.04), cerebrovascular diseases and their sequelae (SHAP +0.02), degenerative diseases of the central nervous system (SHAP +0.02), thyroid diseases (SHAP +0.01), anemias (SHAP +0.01), , psychiatric disorders (SHAP +0.01), transplant recipients and patients depending on respiratory support equipment (SHAP +0.01), and hemorrhagic hematologic diseases (SHAP +0.01).

Legenda: SRE = support respiratory equipment.

Among the variables chosen by the ML model that best discriminated death or hospital discharge, 98% of the predictive power was determined by age, sex, and 15 chronic health conditions (Table 1, Figure 3).

Population risk groups were created by combining these variables in order to turn the results into recommendations that could be easily conveyed to national vaccination program managers.

Age was divided into 8 groups: Age group 0: ≥ 18 < 30 years; Age group 1: ≥ 30 < 40 years; Age group 2: ≥ 40 < 50 years; Age group 3: ≥ 50 < 60 years; Age group 4: ≥ 60 < 70 years; Age group 5: ≥ 70 < 80 years; Age group 6: ≥ 80 < 90 years; Age group 7: ≥ 90 years. Statistical analysis (Kruskal-Wallis test with Post-hoc Dunn test) showed that the incidence of death differed significantly between age groups (p < 0.05) and increased with age (Table 1). Chronic health conditions were transformed into a dichotomous variable: present or absent.

The combination of age groups and other risk factors chosen by the ML model (gender: female or male; presence of at least one of the 15 chronic health conditions: yes or no) resulted in 32 categories for population at risk (Table 2). The incidence of death in the 32 categories was compared using the Kruskal_Wallis test with the Post-hoc Dunn test (significance level of 5%) – categories with no statistical difference between them (p > 0.05) were combined into a single group, resulting in 11 sets of priorities for vaccination (Table 2).

**Table 2:** Eleven (11) vaccination priority groups based on the incidence of death defined by the 32 risk groups.

Group	Incidence of death (%)	Sex	Age group	Chronic health conditions	p	Risk-of-death priority level (Min-Max incidence of death, %)	Risk category-population feature
Group I						Priority 1	
grp32	58.8	1	7	1			Male and female, > 90 years old, with comorbidities.
grp31	50.2	0	7	1	> 0.05	48.7–58.8	Male, 80 to 90 years old, with comorbidities.
grp28	48.7	1	6	1			
Group II						Priority 2	
grp27	38.5	0	6	1			Female, 80 to 90 years old, with comorbidities.
grp30	37.2	1	7	0	> 0.05	34.2–38.5	Male and female, > 90 years old, no comorbidities.
grp29	37.2	0	7	0			Male, 70 to 80 years old, with comorbidities.
grp24	34.2	1	5	1			
Group III						Priority 3	
grp26	28.9	1	6	0			Female, 70 to 80 years old, with comorbidities.
grp23	27.6	0	5	1	> 0.05	25.1–28.9	Female, 80 to 90 years old, no comorbidities.
grp25	26.8	0	6	0			Male, 70 to 90 years old, no comorbidities.
grp22	25.1	1	5	0			
Group IV						Priority 4	
grp20	22.6	1	4	1	< 0.001	22.6	Male, 60 to 70 years old, with comorbidities.
Group V							
grp21	18.5	0	5	0	> 0.05	Priority 5	Female, 70 to 80 years old, no comorbidities.
grp19	18.4	0	4	1		18.4–18.5	Female, 60 to 70 years old, with comorbidities.
Group VI						Priority 6	
grp16	14.1	1	3	1	< 0.001	14.1	Male, 50 to 60 years old, with comorbidities.
Group VII						Priority 7	
grp18	13.1	1	4	0	< 0.001	13.1	Male, 60 to 70 years old, no comorbidities.
Group VIII							
grp15	11.4	0	3	1	> 0.05	Priority 8	Female, 60 to 70 years old, no comorbidities.
grp17	9.9	0	4	0		9.85–11.5	Female, 50 to 60 years old, with comorbidities.
Group IX							
grp12	8.3	1	2	1		Priority 9	Male, 50 to 60 years old, no comorbidities.
grp8	7.9	1	1	1		5.5–8.5	Male and female, 18 to 50 years old, with comorbidities.
grp11	7.8	0	2	1			
grp7	7.4	0	1	1	> 0.05		
grp14	6.4	1	3	0			
grp3	5.7	0	0	1			
grp4	5.5	1	0	1			
Group X							
grp13	3.6	0	3	0		Priority 10	Male and female, 40 to 50 years old, no comorbidities.
grp9	2.5	0	2	0	> 0.05	2.2–3.7	Female, 50 to 60 years, no comorbidities.
grp10	2.2	1	2	0			
Group XI							
grp6	1.4	1	1	0		Priority 11	Male and female, 18 to 40 years old, no comorbidities.
grp2	1.4	1	0	0	> 0.05	0.1–0.2	
grp5	1.2	0	1	0			
grp1	0.1	0	0	0			

Vaccination priority groups defined by Kruskal-Wallis and Post-hoc Dunn statistical tests in the 32 risk groups. p-value > 0.05 indicates no statistically significant difference.

Sex 0: female; Sex 1: male. Age group 0: ≥ 18 < 30 years; Age group 1: ≥ 30 < 40 years; Age group 2: ≥ 40 < 50 years; Age group 3: ≥ 50 < 60 years; Age group 4: ≥ 60 < 70 years; Age 5 group: ≥ 70 < 80 years; Age group 6: ≥ 80 < 90 years; Age group 7: ≥ 90 years. Chronic health conditions: 0 = no chronic health conditions; 1 = presence of at least one of the 15 main chronic health conditions chosen by the ML model.

## DISCUSSION

Vaccination priorities were established by multilateral organizations such as the World Health Organization and governments in different countries. Priorities are based on ethical principles such as Human Well-Being, Equal Respect, Global Equity, National Equity, Reciprocity, and Legitimacy^11^, or on ethical principles^12^set by the Institute of Medicine, which focus on the protection and promotion of public health and socio-economic well-being in the short- and long-term. According to these principles, each individual must be considered and treated with equal dignity and regard. Mitigating healthcare inequalities during the covid-19 pandemic requires explicitly addressing the heavier burden experienced by the most affected populations due to their higher exposure to economic and social inequities, as well as to their unequal access to health. In order to operationalize their fundamental principles, these entities have developed risk-based criteria to define priority populations to be vaccinated, namely: risk of acquiring SARS-CoV-2 infection due to exposure to high virus doses; risk of severe morbidity and mortality; individuals whose disability and death affect the lives and livelihood of other individuals; and risk of transmitting the infection to others^13,14^. Elderly individuals and populations with comorbidities are among the priority populations in all programs implemented worldwide since they are at high risk of morbidity and mortality^13,14^.

The definition of priority vaccination groups based on mortality risk becomes more sensitive and specific if sex, age, and comorbidities are assessed altogether. Who should have priority for vaccination? The 60–65-year-old population without comorbidities or the 39–45-year-old obese population? The current study tries to answer this question, which is of paramount importance to meet the ethical principles adopted worldwide.

The predictive model of death by covid-19 created by ML has been used to develop risk measurement tools to define priority populations to be targeted in public protection policies, such as the ones focused on vaccination. Anuj Tiwari et al. have developed a covid-19 risk of death and infection index, which was determined based on racial and economic inequalities, by using Random Forest machine learning. Populations living in American counties have been categorized into 4 risk levels (very high, high, low, and very low) to help public health authorities and disaster management agencies to develop effective mitigation strategies, especially for the high-risk communities due to their highly vulnerable condition^15^.

Elderly patients and individuals with pre-existing chronic health conditions were highly prevalent in this case study. Their mean age was approximately 60 years and most of them were men; this finding was similar to case studies reported in other countries and in other Brazilian studies^16^. Patients older than 60 years accounted for 15.7% of the Brazilian population, as well as for 51.1% of hospital discharges/deaths observed in the investigated sample^1^.

Hypertensive diseases, diabetes mellitus, obesity, cancer, heart failure, asthma, and obstructive pulmonary diseases were the chronic health conditions most often observed in the current study. These chronic health conditions were similar to the ones reported for the New York City area^16^, China^17^ and Brazil^18^.

Mortality rate increased with the patients’ age. Overall hospital mortality was of 17.9% (8,823 patients), 16,627 hospitalized patients (33.8%) required intensive care, and 9,411 (19.1%) of them required invasive mechanical ventilation; these results were similar to the ones reported in other studies^17,18^.

The number of chronic health conditions per patient was higher than that observed in other studies conducted in Brazil^18^, 1.97 ± 1.85 (mean ± SD). This finding can be attributed to the quality of data analyzed in this study, since data collection was carried out by coders who were specially trained for this task, possibly increasing the number of properly collected data.

The 17 most relevant independent variables defined by the SHAP method, and used to determine patients’ risk of death, are also reported in the literature. Among them are: age^19,20^, male sex^21^, obesity^22^, diabetes^23^, chronic renal failure^24^, chronic arterial hypertension^22,24,25^, myocardial and cardiac valvular diseases and arrhythmias^20,22,24^, neoplasms^20,24,27^, chronic respiratory diseases^20,24,25^, cerebrovascular disease and its sequelae^20,22,24^, thyroid diseases^28,29^, anemias^30^, degenerative diseases of the central nervous system^24,25^, psychiatric diseases^31^, and transplant recipients^32^ (Table 1).

Priority covid-19 vaccination population groups defined in this study (Table 2) will enable countries that do not have specific information about their populations to further refine priorities capable of saving lives.

Large sample size and quality of collected data are the strongest features of our study. On the other hand, the study has several limitations. We understand that other important variables can be used to create priority groups for vaccination. These variables are associated with the type and intensity of individuals’ exposure to risks, such as their occupation, social inequities, and others. However, as we did not have this information available, they were not included in this study. Furthermore, the proportion of hospitalizations in the public healthcare system was lower (33.47%) in the investigated sample than the one observed in Brazil (57.7%)^2,3^. The population in the private healthcare system comprises workers from Brazilian companies or individuals who can afford a private healthcare insurance; they account for 22.3% of the Brazilian population^1,4^. The population in the public system comprises several unemployed workers, but also employed and low-income workers. These differences in income, living conditions, and access to treatment were not evaluated in our study. It is necessary to conduct the external validation of this in-hospital predictive model for other populations.

Our study was based on data of thousands of covid-19 patients. Data were collected throughout the pandemic at a global epicenter (Brazil), a fact that led to relevant findings to the current context. Results were based on rigorous machine learning analyses powered by a robust sample comprising patients with laboratory-confirmed SARS-CoV-2 infection.
